# Quantitative assessment of chemotropism in pollen tubes using microslit channel filters

**DOI:** 10.1063/1.5023718

**Published:** 2018-03-30

**Authors:** Naoki Yanagisawa, Tetsuya Higashiyama

**Affiliations:** 1 Division of Biological Science, Graduate School of Science, Nagoya University, Furo-cho, Chikusa-ku, Nagoya, Aichi 464-8602, Japan; 2 Institute of Transformative Bio-Molecules (ITbM), Nagoya University, Furo-cho, Chikusa-ku, Nagoya, Aichi 464-8602, Japan

## Abstract

We present a semi-*in vitro* chemotropism assay that can be used to evaluate the chemoattractant effect of diffusible plant signaling molecules on growing pollen tubes. We constructed an array of microslit channels in a microfluidic device that prevented the passage of randomly growing pollen tubes but permitted ones that are responsive to the chemoattractant. Depending on the microslit channel size, 80%–100% of the randomly growing *Torenia fournieri* pollen tubes were excluded from reaching the source of the attractant. Thus, the selection of pollen tubes that are capable of responding to chemoattractants from a mixed population can be realized using this platform.

## INTRODUCTION

In flowering plants, the main role of pollen tubes is to deliver the nonmotile sperm cells to ovules for fertilization. During this process, pollen tubes are guided to the target plant tissue by sensing multiple signaling cues from their surroundings.^1^ Semi-*in vitro* pollen tube chemotropism assays are a fundamental method to examine pollen tube guidance.^2^ In this method, the dissected style of a pollinated pistil is co-cultured with an ovary or ovules in growth medium, and pollen tubes emerging from the cut end of the style and approaching the female gametophyte are observed using a microscope.^3,4^ Several important discoveries in the field of plant reproduction have been made using this technique in the past decade, such as the identification of pollen tube attractants^5,6^ and their receptors,^7,8^ and of a key molecule that enhances the competency of pollen tubes to respond to attractants.^9^

**FIG. 1.**
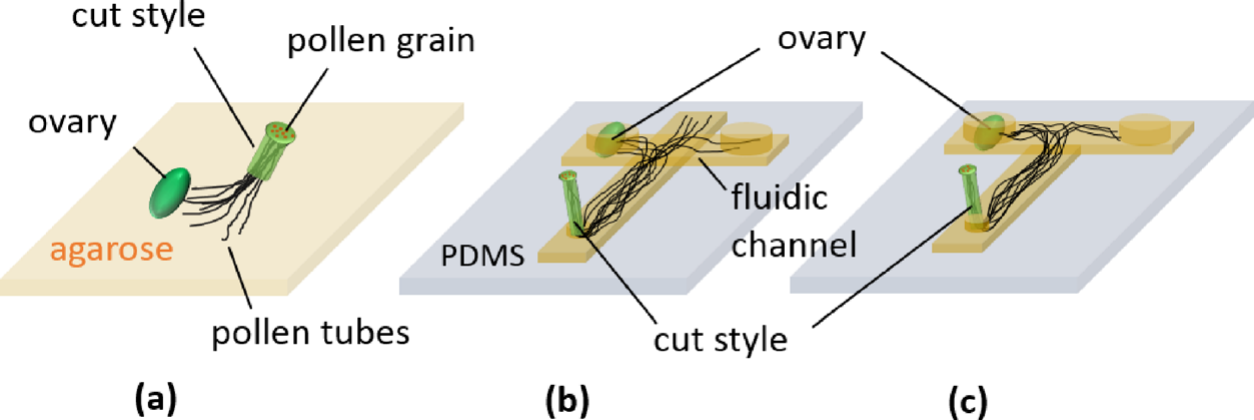
Semi-*in vitro* pollen tube chemotropism assay. (a) Conventional method in which a cut style of a pollinated pistil and a plant ovary are placed on an agarose plate. The ovary wall is usually removed, and a naked ovary (ovules on the placenta) is used for the assay. (b) Microfluidic approach using a cross-shaped and (c) T-shaped channel. The assay result is determined by quantifying the number of pollen tubes entering the channel segment where an ovary is placed.

In a typical semi-*in vitro* chemotropism assay, pollen tubes are grown on agarose medium in a Petri dish and assessed based on whether they have elongated in the direction of the female tissue [Fig. 1(a)]. This type of assay is particularly effective when chemoattracted pollen tubes clearly change their growth direction to the target tissue and exhibit a distinct growth behavior compared to randomly growing ones. However, due to the qualitative nature of this method, differences in the experimental outcomes of a series of assays may not be discernable and are open to subjective interpretation. There is therefore a need to develop a quantitative assay for assessing pollen tube guidance.

Conducting a quantitative analysis of pollen tube guidance requires a clear definition of an attracted pollen tube. One promising approach for selecting chemoattracted pollen tubes involves a microfluidic device, which comprises small fluidic conduits that can be used as miniature growth chambers with precisely defined geometries. Microfluidic platforms have been employed to analyze chemotactic responses of various cell types, including bacterial cells,^10,11^ cancer cells,^12,13^ and multicellular organisms such as *Caenorhabditis elegans.*^14,15^

To date, several microfluidic devices have been developed to perform quantitative analyses of pollen tube guidance. The first reported microfluidic-based pollen tube chemotropism assay used a cross-shaped channel [Fig. 1(b)]. *Arabidopsis thaliana* ovules were placed in one of the side channels, and the pollinated style was placed in the central channel. Attracted pollen tubes were then quantified based on their entry into the channel containing the ovules.^16^ Using the same approach, pollen tube guidance in *Torenia fournieri* was demonstrated in a microfluidic device with cross-shaped^17^ and T-shaped channels^18^ [Fig. 1(c)]. All microfluidic-based assays reported to date consider pollen tubes that enter the ovary placed channel as responsive to the chemoattractant, even though not every pollen tube has a competency to respond to attractants.^9^ Therefore, it is likely that simple geometries, such as the cross-shaped or T-shaped channels described above, allow some pollen tubes to randomly grow into the ovules placed channel, resulting in false positives.

Previously, Agudelo *et al.* demonstrated pollen tube elongation inside of microchannels with various geometries.^19^ In this work, we have developed a microslit channel array (2–16 *μ*m in width and 5 *μ*m in height) that selectively allows the passage of chemoattracted pollen tubes (approximately 8 *μ*m in diameter) but mostly prevents randomly growing ones from approaching the source of the attractants. Using this device, we conducted a pollen tube chemotropism assay for *T. fournieri* and characterized the selectivity of the chemoattracted pollen tubes to an ovary.

## MATERIALS AND METHODS

### Device preparation

**FIG. 2.**
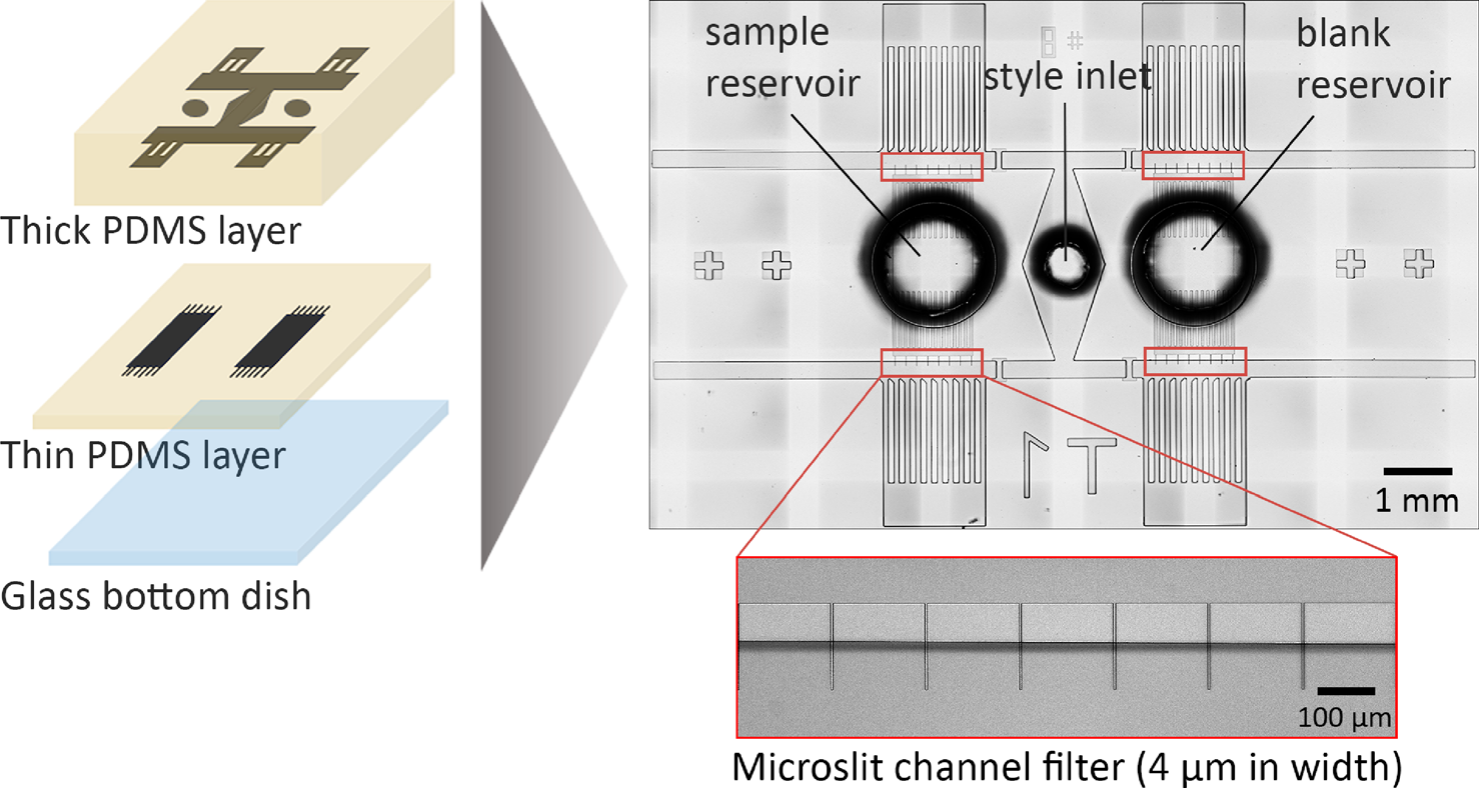
Design and preparation of the microfluidic device. A thin (bottom) PDMS layer with microslit channels is aligned and covered with a thick (top) PDMS layer. The constructed device is placed on a glass bottom dish for microscopy analysis. Channel height: 90 *μ*m (top layer) and 5 *μ*m (bottom layer).

The microfluidic device developed for this work is composed of two poly-dimethylsiloxane (PDMS) layers: a thick PDMS layer with fluidic channels for pollen tube growth and a thin PDMS layer with an array of microslit channels that serve as a filter for isolating chemoattracted pollen tubes (Fig. 2). The height of the microslit channels created on the thick and thin layers is 90 *μ*m and 5 *μ*m, respectively, unless otherwise specified. The PDMS molds for the top and bottom layers were fabricated separately on silicon wafers by spin-coating negative photoresist (SU-8 3005 for the bottom layer, 3010 and 3050 for the top layer; Microchem Corp.) The microslit channel patterning onto the photoresist layers was conducted using a maskless lithography system (DL-1000; Nano System Solutions, Inc.). While the top PDMS layer was made by simply pouring a prepolymer mixture of PDMS (Sylgard 184; Dow Corning) onto the mold, the bottom layer was prepared by spin-coating the same prepolymer mixture of PDMS. After curing at 65 °C for 90 min, the top PDMS layer was punched to form inlets, and both layers were plasma treated in air and subsequently assembled with the assistance of a custom-made desktop aligner. Finally, the constructed PDMS device was heated at 65 °C for another 60 min to completely seal the microchannels.

### Semi-*in vitro* pollen tube chemotropism assay preparation

Pollen tube growth medium described by Higashiyama *et al.*^3^ was prepared in 1% (w/v) ultra-low melting point agarose (Type IX-A; Sigma) and introduced into the microslit channels using a vacuum filling method.^20^ The agarose was solidified by cooling the device at 5 °C for 5 min. Wild-type *Torenia fournieri* “blue and white” flowers were cultivated at 28 °C under long-day conditions (16 h light/8 h darkness). To grow pollen tubes in the flow channels, the pistil was hand-pollinated, and the style was cut to a length of 15 mm before being inserted into the inlet of the device. An ovary was immersed in the agarose filled sample reservoir after its wall was manually removed with a blade. Subsequently, the device was kept in an incubator at 28 °C for 24 h in darkness.

### Time-lapse imaging of pollen tube growth

Time-lapse images of *T. fournieri* pollen tube growth were captured using an inverted microscope (IX-83; Olympus) equipped with a spinning-disk confocal system (CSU-W1; Yokogawa Electric) and an electron multiplying charge-coupled device digital camera (iXon3; Andor Ltd.). Images were acquired using the microscopy automation software MetaMorph (Molecular Devices).

### Scanning electron microscopy (SEM) image acquisition

The SEM images of microchannel structures were captured using a digital microscope (VHX-1000 with VHX-D500/D510 lens; Keyence).

### Visualization of a chemical concentration gradient

To visualize the formation of a chemical concentration gradient in the assay area, we used Alexa fluorophore 488 conjugated dextran, 10 000 MW (Thermo Fisher Scientific) which mimics the size of a pollen tube attractant peptide, LURE1. The fluorescent dye was excited by a 488 nm laser (Coherent), and fluorescence images were captured every hour for 24 h by an electron multiplying charge coupled device digital camera (iXon3; Andor Ltd.). The intensity of the obtained fluorescence images was later quantitated using an in-house written MATLAB code.

## RESULTS AND DISCUSSION

### Design of a semi-*in vitro* pollen tube chemotropism assay device

**FIG. 3.**
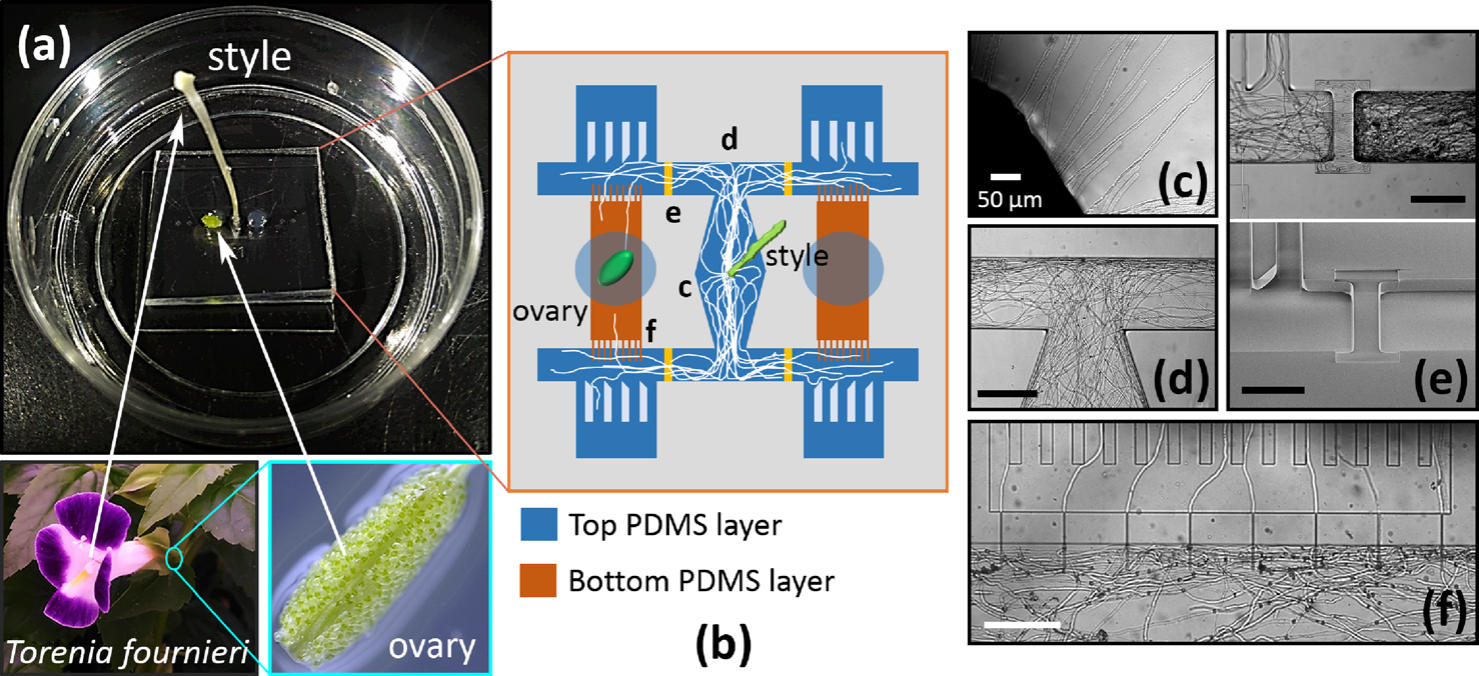
Device setup and pollen tube growth in a microfluidic environment. (a) Optical image of the microdevice setup. A cut style of a pollinated pistil (length: 15 mm) and an ovary of *T. fournieri* were placed in the device. (b) Schematic of the microchannel design. (c) Pollen tubes emerged from the cut end of the style. (d) T-channel section showing that the pollen tubes elongate to either the left or right without preference. (e) Shallow channel segment created to reduce the number of pollen tubes entering the assay area (upper image) and SEM image showing its channel geometry (lower image). In Multimedia view, the time stamp shows the time after pollination. Scale bar, 100 *μ*m. (f) Pollen tubes passing through the microslit filter (width: 4 *μ*m). Scale bar, 200 *μ*m unless specified otherwise. Multimedia view: https://doi.org/10.1063/1.5023718.1
10.1063/1.5023718.1

In Fig. 3(a), we present the experimental setup for the pollen tube chemotropism assay using the proposed microfluidic device. By placing a pollinated and dissected pistil in the inlet of the device, the pollen tubes that germinated at the stigma elongated through the style and subsequently entered the fluidic channel [Fig. 3(c)]. When the pollen tubes reached the T-shaped channel junction [Fig. 3(d)], they grew either to the left or right without preference, indicating that the presence of an ovary in the sample reservoir did not seem to affect their growth direction. The number of pollen tubes which can enter the assay area depends on the amount of pollen grains germinated on the stigma. Because the flower is manually pollinated, the number of pollen tubes that grow inside of the device is not preciously controllable. Therefore, there can be a case where a substantially large number of pollen tubes enter the assay area and totally occupy the assay space. In this situation, some pollen tubes may proceed into the slit-channels regardless of their responses to the chemoattractants simply because no other spaces are available for them to elongate. To prevent such a case from happening, we prepared shallow channel segments (channel height: 13 *μ*m) to limit the number of pollen tubes that can enter the assay areas [Fig. 3(e) (Multimedia view)]. As can be seen, although more pollen tubes reached the shallow channel segment as time passed, it became more difficult for them to enter the assay area due to a gradual blockage of the shallow segment by the pollen tubes that had already crossed it.

Another feature of this device is that it consists of two PDMS layers in which the pollen tubes elongate through the channels prepared on the top layer, while the entrance to the microslit channels is located on the bottom layer. Therefore, the pollen tubes first need to grow downwards (from the top to the bottom layer) to pass through the microslit channel filter. This channel geometry was highly effective at preventing randomly growing pollen tubes from advancing towards the ovary [Fig. 3(f), supplementary material Movies 1 and 2]. The detailed microchannel structure of the assay area is presented in supplementary material Fig. 1.

**FIG. 4.**
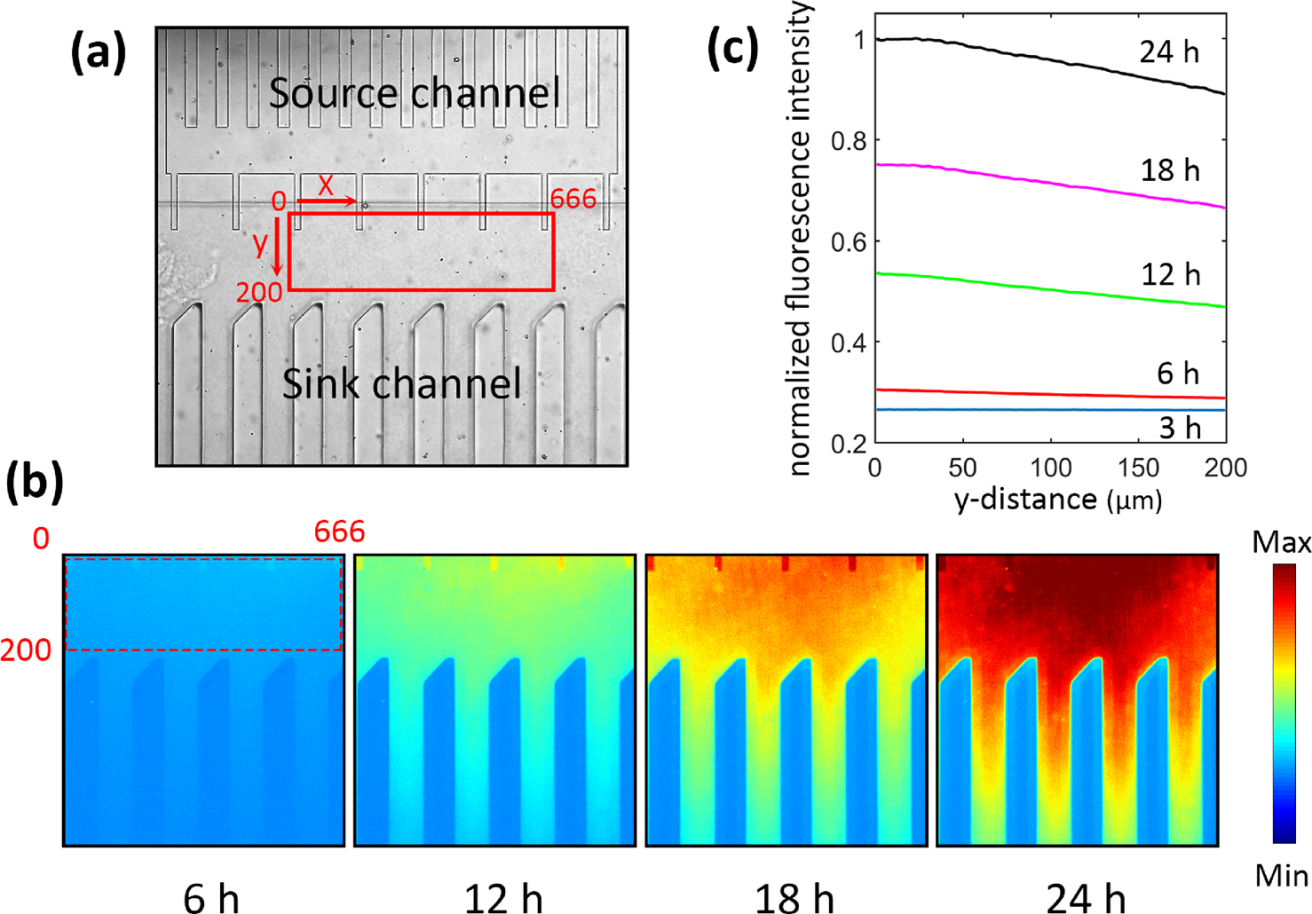
Evaluation of a concentration gradient of Alexa 488-conjugated 10 kDa dextran generated in the assay area. (a) A microscopy image of the assay area with a 16-*μ*m wide slit-channel filter in the device. The area indicated by a rad colored box (666 *μ*m × 200 *μ*m) was evaluated for the fluorescence intensity measurement. (b) Color maps showing the fluorescence intensity profile of the dye obtained at 6, 12, 18, and 24 h after sample introduction. (c) Normalized fluorescence intensity plots obtained by averaging the intensity along the x-axis (from 0 to 666 *μ*m) and scanning along the y-direction (from 0 to 200 *μ*m). The fluorescence intensity in the slit-channel areas was excluded in this analysis considering that the optical path length in these areas is different from the rest of the region.

We also visualized the formation of a chemical gradient in the assay area [Fig. 4(a)] by introducing Alexa fluorophore-conjugated 10 kDa dextran, which is similar to the size of a pollen tube attractant peptide,^5^ LURE1. As shown in Fig. 4(b), the fluorescent dye was diffusing through the microslit channels, generating its gradient in the assay area. Although the use of a dye does not illustrate the actual chemoattractant gradient that can be generated by the plant ovary, we found that a gradient of the dye gradually disappeared near the entrance to the microslit channels within 24 h [Fig. 4(c)]. We assume that such a loss of the gradient may not happen when we use a plant ovary as a source of attractants considering that an ovary keeps secreting attractants to the sample reservoir.

### Characterization of microslit channel filters

While the diameter of an unconstricted *T. fournieri* pollen tube is approximately 8 *μ*m, we previously observed that these tubes are capable of elongating through a 1-*μ*m wide gap when forced to elongate toward the micro-gap region.^21^ In the device developed in our current work, on the other hand, pollen tubes could grow in any direction. However, those that respond to signaling cues from the plant ovary will change their growth direction towards the higher concentration of the chemoattractant. In this situation, we hypothesized that those pollen tubes would likely to penetrate the microslit filter despite its narrow width. By contrast, the pollen tubes that are not responsive to chemoattractants would not attempt to enter such a physically confined space. Thus, pollen tubes that are capable of responding to chemoattractants could be selectively isolated from a population.

**FIG. 5.**
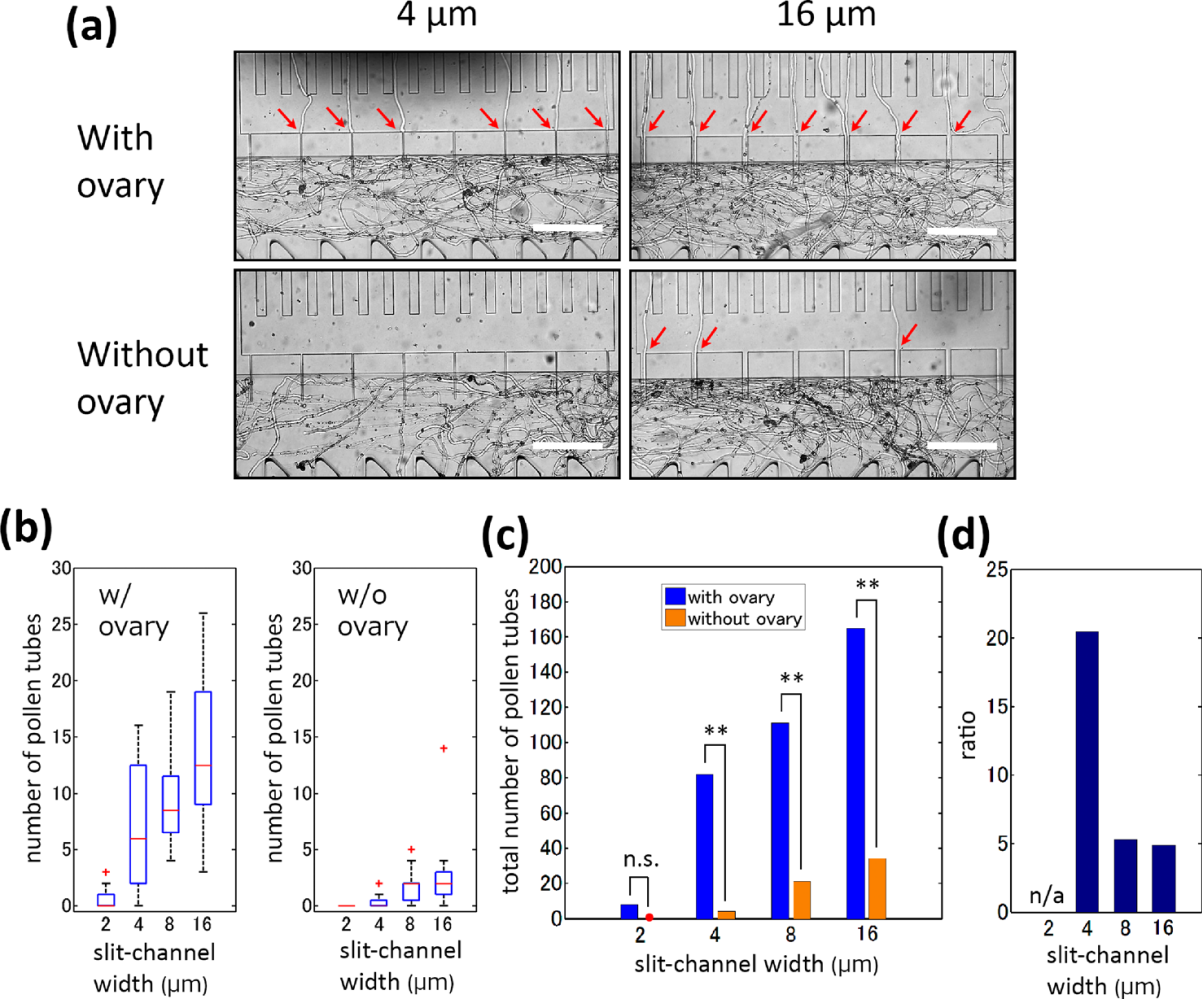
Summary of semi-*in vitro* pollen tube chemotropism assay results. (a) Representative microscopy image of the assay areas captured at 24 h after pollination. Microslit filters (4 *μ*m and 16 *μ*m wide) were located in front of the sample and blank reservoirs. Red arrows indicate pollen tubes that passed through the filter. Scale bar, 200 *μ*m. (b) Box plots showing the number of pollen tubes passing through each microslit channel filter (n = 12 replicates). The red bar denotes the population median, and boxes are the 25th and 75th percentiles. Outliers are set when the data points are outside of 1.5 × IQR (interquartile range). (c) Total number of pollen tubes passing through the microslit filter after 12 replicates. ** indicates significant differences (p < 0.01, Wilcoxon signed-rank test); n.s. (not significant) when p > 0.05. (d) Ratio of the experimental results obtained in the presence and absence of an ovary, which is shown in (c).

To test this idea, we evaluated the efficiency of four different microslit channel widths (2, 4, 8, and 16 *μ*m) to selectively isolate pollen tubes that respond to the chemoattractant. Representative images obtained using 4-*μ*m and 16-*μ*m filters are presented in Fig. 5(a). The results of the pollen tube chemotropism assay were evaluated by quantifying the number of pollen tubes that had passed through the microslit channels at 24 h after pollination. Because the number of pollen tubes that reached the assay areas was occasionally very low, presumably due to the small number of pollen grains transferred to the stigma, the assay results were only considered to be valid when 10 or more pollen tubes were observed in all four assay areas at 24 h after pollination. The number of pollen tubes passing through the microslit filters in each device and the sum of such pollen tubes obtained from 12 devices (replications) are shown in Figs. 5(b) and 5(c), respectively. Microslit filters with a channel width of 4–16 *μ*m preferentially permitted the chemoattracted pollen tubes to grow towards the plant ovary. In addition, the use of a 2-*μ*m filter eliminated 100% of the randomly growing pollen tubes. However, we found that even when an ovary was placed in the sample reservoir, the number of pollen tubes crossing such narrow filters was very low (0.8 per device), resulting in a statistically non-distinguishable outcome between the positive and negative controls.

We defined the selectivity of the chemoattracted pollen tubes as the ratio of the number of pollen tubes crossing the filter in the presence of a plant ovary in the reservoir to that in the absence. This was determined based on the results shown in Fig. 5(c) and summarized in Fig. 5(d). A comparison of the results obtained using the 8-*μ*m and 16-*μ*m microslits showed that the number of pollen tubes that crossed the microslit channels was 49% greater for the wider channels in the presence of an ovary and 62% greater in the absence of an ovary. In both cases, the probability of pollen tubes passing through the filter was greater for those with 16-*μ*m microslits simply because the wider slit width could accommodate more pollen tubes. The previously reported cross-shaped [Fig. 1(b)] and T-shaped channel [Fig. 1(c)] microdevices do not contain any physical barriers, such as the proposed microslit channels, separating the pollen tubes from the ovary. Our results indicate that in such a condition, randomly growing pollen tubes can easily proceed to a particular channel regardless of their attraction to signaling cues from a plant ovary. As shown in Fig. 5(d), selectivity of chemoattracted pollen tubes was similar for assays conducted using 8-*μ*m and 16-*μ*m microslits, i.e., 5.3 and 4.9, respectively. By contrast, the selectivity of the device with a 4-*μ*m microslit filter was significantly increased (i.e., 20.5) mostly because the number of randomly growing pollen tubes that passed through the filter was substantially reduced. Our results suggest that it is effective to use a channel that is slightly narrower than the diameter of the pollen tube (e.g., 4 *μ*m in the case of *T. fournieri* pollen tubes), whereas a channel that is too narrow (e.g., 2 *μ*m) prevents entry of pollen tubes altogether.

## CONCLUSIONS

The purpose of this work was to develop a bioassay platform that allowed us to quantitatively evaluate chemotropism in pollen tubes in response to signaling cues produced by a plant ovary. We used microslit channels to spatially separate the chemoattracted pollen tubes from randomly growing ones. The best selectivity of the chemoattracted pollen tubes was achieved when the width of the microslit channel was approximately half of the cylindrical diameter of *T. fournieri* pollen tubes. Ovules in the ovary are believed to provide multiple directional cues from different ovular cells.^1^ Various mutants of the model plant species *Arabidopsis* show impaired pollen tube guidance; however, these defects are challenging to quantify. The microfluidic device presented here will be useful for quantifying ovular attraction by ovular mutants of *T. fournieri*, which can be easily produced using CRISPR/Cas9 genome editing.^22^ While the device presented here uses living ovary tissue as the source of the pollen tube attractant, there is also a strong need for a high-throughput assay to evaluate the activity of pollen tube attractants such as the LURE peptides^5,6^ and other candidate attractant molecules. We are currently developing a microfluidic-based assay that can assess the activity of purified chemoattractants in a high-throughput manner and we plan to report the details of this assay in the future.

## SUPPLEMENTARY MATERIAL

See supplementary material for the microchannel structure of a pollen tube chemotropism assay area. Movie 1 and Movie 2 show representative assay results in the presence and absence of a plant ovary, respectively. An 8-*μ*m wide microslit channel filter was used. The time stamp shows the time after pollination. Scale bar, 100 *μ*m.
